# Biosensors Based on Plasmonic Spoon-Shaped Platforms as a Point-of-Care Tool for *Escherichia coli* Detection

**DOI:** 10.3390/bios16070371

**Published:** 2026-07-08

**Authors:** Francesco Arcadio, Alessandro Capo, Alessia Calabrese, Chiara Marzano, Mimimorena Seggio, Rosalba Pitruzzella, Federica Passeggio, Shahab Bashir, Muhammad Shoaib, Carla Zannella, Anna De Filippis, Giuseppe Portella, Luigi Zeni, Nunzio Cennamo

**Affiliations:** 1 Department of Engineering, University of Campania Luigi Vanvitelli, Via Roma 29, 81031 Aversa, Italy; francesco.arcadio@unicampania.it (F.A.); chiara.marzano@unicampania.it (C.M.); rosalba.pitruzzella@unicampania.it (R.P.); federica.passeggio@unicampania.it (F.P.); luigi.zeni@unicampania.it (L.Z.); 2 Italian National Research Council, Institute of Food Sciences, URT-Napoli, 80100 Naples, Italy; alessandro.capo@cnr.it; 3 Department of Biology, University of Naples Federico II, Via Cinthia, 80126 Naples, Italy; alessia.calabrese@unina.it; 4 Department of Engineering, Pegaso University, 80143 Naples, Italy; mimimorena.seggio@unipegaso.it; 5 Department of Woman, Child and General and Specialized Surgery, University of Campania “Luigi Vanvitelli”, 80138 Naples, Italy; shahab.bashir@unicampania.it (S.B.); muhammad.shoaib1@unicampania.it (M.S.); carla.zannella@unicampania.it (C.Z.); anna.defilippis@unicampania.it (A.D.F.); 6 Department of Translational Medical Sciences, University of Naples Federico II, Via Pansini, 80131 Naples, Italy; giuseppe.portella@unina.it; 7 UOC Virology and Microbiology, University Hospital “Luigi Vanvitelli”, 80138 Naples, Italy

**Keywords:** optical biosensors, spoon-shaped waveguides, surface plasmon resonance (SPR), *Escherichia coli* detection, plastic optical fibers (POFs), point-of-care test (POCT)

## Abstract

The *Enterobacteriaceae* family is a significant source of foodborne pathogens and represents a severe threat to human and animal health. These bacteria can penetrate the dairy supply chain through direct contact with cattle and the livestock environment and can survive production processes. *Escherichia coli* (*E. coli*), one of the most diffuse bacteria in raw and processed milk, exposes consumers to the risk of contaminated milk. As a result of this exposition, several milk-borne illness outbreaks have been reported worldwide, underscoring the urgent need for effective detection and prevention measures. Conventional analysis methods are effective but have significant limitations, including the requirement of pre-treatment and pre-enrichment steps. Thus, the need for advanced detection techniques that can accurately identify these pathogens without pre-treatment steps is critical. In this work, a proof-of-concept biosensor based on a spoon-shaped optical biochip was developed to detect *E. coli* via surface plasmon resonance (SPR) phenomena and was combined with a polyclonal antibody layer against *E. coli* as a molecular recognition element (MRE). The proposed label-free biosensing strategy, achieved by exploiting simple SPR spoon-shaped biochips, exhibits a remarkable detection limit (6.8 colony-forming units, CFU/mL) and high specificity towards other interfering bacteria belonging to the *Enterobacteriaceae* family. In addition, tests on commercial milk samples were carried out, achieving recovery values of 95% and 102% for whole milk and infant milk, respectively. The proposed spoon-shaped biosensor enables label-free biosensing without the need for microfluidic systems. It provides a rapid response (10 min), paving the way for its use as a point-of-care test (POCT) in real-world settings.

## 1. Introduction

*Enterobacteriaceae* is a family of Gram-negative and non-spore-forming bacteria responsible for food contamination. Belonging to the *Enterobacteriaceae* family, the most common pathogen is *Escherichia coli* (*E. coli*), frequently found in food- and milk-borne illness outbreaks [1,2]. This microorganism was primarily classified based on the serologic identification of O (lipopolysaccharide, LPS) and H (flagellar) antigens and then through the virulence components in pathogenic strains [3]. It is a rod-shaped bacterium that lives as a commensal resident in the gastrointestinal tracts of warm-blooded animals, including humans. Nevertheless, it represents one of the most frequent causes of a wide range of common infections in humans and animals through transmission via the fecal–oral pathway and by consuming contaminated food or drink. The consumption of raw food, such as raw drinking milk (RDM), has increased consumer health risks due to *E. coli* and other foodborne pathogens. Several outbreaks due to *E. coli* presence, in its enteropathogenic and enterohemorrhagic (EHEC) forms, when raw drinking milk, inadequately pasteurized milk, not-well-preserved milk, and other foods have recently been reported [4,5]. The main *E. coli* pathotype responsible for milk outbreaks is the Shiga toxin-producing *E. coli* (STEC), a causative agent of hemorrhagic colitis and hemolytic uremic syndrome. STEC infections in humans are obtained through ingesting infected foods [6]. The *E. coli* serotype O157:H7 is the STEC most closely linked to human food-related diseases. The global rise in STEC serotypes other than O157 (O111, O26, O145, and O103) has been associated with outbreaks of foodborne illness, according to recent studies [7]. In the dairy supply chain, milk-borne epidemics linked to STEC O157:H7 have been documented in Finland [4], Germany [4], and the United States [8,9].

The main methods for detecting and quantifying *E. coli* in food products include plate counting, immuno-magnetic separation, flow cytometry, polymerase chain reaction (PCR), and chromogenic–fluorogenic substrate technology [10,11,12,13,14]. These methods often require sample pre-treatment, and in many cases, the low level of *E. coli* in foods necessitates a pre-enrichment step to enhance the sensitivity, thereby increasing the time for analysis [15,16]. On the contrary, detection systems based on optical sensing techniques are emerging as the most promising for *E. coli* bacteria monitoring [17]. These methods include several strategies, for example, those based on absorption, fluorescence, reflection, and chemiluminescence [18,19,20,21,22]. Along this line of research, Zhao et al. [23] presented a surface-enhanced Raman scattering (SERS) platform based on graphene oxide–gold nanostar tags coupled to aptamer-functionalized magnetic capture probes for the simultaneous detection of *E. coli* and *Staphylococcus aureus*.

The optical biosensors based on surface plasmon resonance (SPR) and localized surface plasmon resonance (LSPR) phenomena were possible alternative technologies for *E. coli* detection. Indeed, plasmonic biosensors produce real-time results by exploiting the detection of slight variations in the refractive index at the interface between a functionalized metal (e.g., gold) nanofilm or nanostructure and a dielectric medium, following the interaction between the analyte and the molecular recognition element (MRE) [24,25].

In this context, different plasmonic solutions were developed to detect *E. coli*. Wang et al. [22] developed a portable SPR bioanalyzer in which the experimental setup consisted of an integrated biosensor and a microfluidic cell with a three-way solenoid valve. The antibody was immobilized on the gold surface, allowing a Limit of Detection (LoD) of 1.87 × 10^3^ colony-forming units (CFU)/mL. In previous studies, Song et al. [26] and Petronella et al. [27] proposed an LSPR sensing strategy to amplify the signal from gold nanorods, reaching an LoD of 10 CFU/mL and 8.4 CFU/mL, respectively. Additional plasmonic sensor configurations for *E. coli* detection based on optical fibers were developed. For instance, Kaushik et al. [28] proposed an SPR immunosensor modified with molybdenum disulfide (MoS_2_) nanosheets using monoclonal anti-*E. coli* antibodies as the MRE, and the obtained LoD value was 94 CFU/mL. Zhou et al. [29] developed an SPR optical fiber sensor to detect *E. coli* applied to water and juice matrices.

Recently, SPR probes implemented in spoon-shaped waveguides have been developed to achieve different sensitivities [30]. Specifically, a polymeric multimode optical waveguide, characterized by a spoon-shaped geometry, is proposed to excite different SPR phenomena by changing the sensitive area and the position of the source and detector connected to the spoon-shaped waveguide. A gold nanofilm covers the spoon-shaped waveguide to achieve the SPR phenomena in two distinct sensing regions that can be exploited to obtain biosensors, such as the planar one on the spoon’s neck and a concave one on the bowl. Therefore, the sensor’s performance can be conveniently adjusted based on the selected sensing region and experimental setup, as reported by Cennamo et al. [30]. The capability to excite different plasmonic sensitivities via the same SPR platform can be used to implement sensor arrays or to achieve biosensors with ultra-wide detection ranges, and both characteristics are helpful for bio/chemical applications. Moreover, the bowl region presents a built-in measuring cell useful for on-site measurements without microfluidic systems [30]. The peculiar geometry of the bowl-sensitive area promotes light scattering, thus improving plasmonic performances [30].

In general, several methodologies in SPR probes are used to monitor interactions between MREs and large analytes (micrometric in size), such as microparticles, bacteria, and cells. For this purpose, different strategies in SPR platforms based on optical waveguides can be adopted, such as improving the optical penetration depth by increasing the working wavelength to the mid-infrared [31] or optical telecommunication window [32]; alternatively, optical buffer layers with refractive indices greater than that of the core of the waveguide can be used to improve the performances [33], like in the case of SPR sensors based on plastic optical fibers (POFs) employed in microplastic [34] and *Brucella abortus* detection [35].

In the proposed work, the distinct geometry of the bowl-sensitive area enhances light scattering of the propagated light, thereby improving the optical penetration depth in the visible range and, consequently, the plasmonic performance [30]. More specifically, an optical penetration depth of 800–900 nm can be achieved by exploiting the evanescent field of the specialty waveguide and plasmonic phenomena induced by a continuous gold nanofilm, thereby enabling the monitoring of interactions between receptor layers and micron-sized analytes.

In this work, the bowl area of the SPR sensor based on spoon-shaped waveguides was functionalized with polyclonal antibodies (pAbs) raised against *E. coli* (anti-*E. coli* pAbs) and was used to determine the presence of different strains of *E. coli* (RB1791 and ATCC 11228) in water solutions, such as in PBS, 5% skim milk, and culture medium. Furthermore, selectivity tests were performed to evaluate the possible cross-reactivity of anti-*E. coli* antibodies with other bacteria, namely *Salmonella enterica* (*S. enterica*), *Pseudomonas aeruginosa* (*P. aeruginosa*), *Klebsiella pneumoniae* (*K. pneumoniae*), *Proteus mirabilis* (*P. mirabilis*), and mixed cultures.

In addition, experimental tests were conducted on commercial whole milk and infant milk to assess the usability of the proposed spoon-shaped biosensor in a real-world setting.

## 2. Materials and Methods

### 2.1. Chemicals

α-Lipoic acid, N-hydroxysuccinimide (NHS), N-(3-dimethylaminopropyl)-N′-ethylcarbodiimide hydrochloride (EDC), ethanolamine (2-aminoethanol), 10 mM phosphate-buffered saline at pH 7.4 (PBS), 50 mM 2-(N-morpholino)ethanesulfonic acid at pH 5.5 (MES), and skim milk powder were obtained from Merck-Sigma (Darmstadt, Germany). The secondary antibody, a goat polyclonal-to-rabbit IgG-HRP conjugate, was obtained from Abcam (Cambridge, UK). *E. coli* (RB791 and ATCC 11228), *S. enterica*, *K. pneumoniae*, *P. mirabilis*, and *P. aeruginosa* strains were part of our bacteria collection. The antibodies against *E. coli* RB791 were produced by Covalab SAS (Bron, France). The antibodies against the classical swine fever virus (anti-CSFV) were purchased from Alpha Diagnostic (San Antonio, TX, USA) (ref. # CSFE21-S), and the nProtein A Sepharose 4 Fast Flow was purchased from Cytiva (Washington, DC, USA).

### 2.2. Antibody Production and Characterization

Antibodies against *E. coli* were produced by Covalab SAS. Briefly, *E. coli* (antigen) was inoculated intradermally in two rabbits, and during the immunization period (120 days), the sera were analyzed by an indirect ELISA test to evaluate the antibody titer. After the immunization period, the IgG anti-*E. coli* antibody was isolated from the serum according to Doulamis et al. [36]. In brief, 1.0 mL of rabbit serum, previously diluted 1:1 in 20 mM sodium phosphate buffer at pH 7.0, was loaded onto a Protein A column. The IgG-containing fractions were then eluted using 0.1 M citrate buffer at pH 3.0 and promptly neutralized with 1 M sodium borate buffer at pH 8.5. The homogeneity of the purified IgGs was evaluated by sodium dodecyl-sulfate polyacrylamide gel electrophoresis (SDS-PAGE), and the final concentration was determined by the absorption value at 278 nm.

### 2.3. Bacteria Preparation

The following Gram-negative reference strains were used in this study: *E. coli* ATCC 11228 and *E. coli* RB791 (a non-pathogenic strain of *E. coli*), *S. enterica* serovar Typhimurium ATCC 14028, *P. aeruginosa* ATCC 13388, *K. pneumoniae* ATCC 10031, and a clinical isolate of *P. mirabilis*. All strains were routinely maintained on Luria–Bertani (LB) broth. Single colonies from overnight LB agar plates were inoculated into LB broth and incubated at 37 °C with shaking (150–200 rpm) to obtain starter cultures. These were used to prepare pre-cultures that were grown further until reaching an optical density at 600 nm (OD_600_) of approximately 0.5, corresponding to the mid-exponential phase. At this stage, cultures were subjected to serial dilution and plating for OD–CFU calibration and preparation of defined mono- and co-cultures. More specifically, *E. coli* ATCC 11228/*S. enterica* and *E. coli* ATCC 11228/*K. pneumoniae* mixed cultures were prepared.

*E. coli* RB791 bacteria were suspended and diluted in PBS (pH 7.4) and 5% non-fat dried milk. Samples were obtained starting from the overnight culture by a serial dilution (1:10), in a range from 10^6^ to 10^1^ CFU/mL. The prepared serial dilutions were assessed by the plate counting method (three Petri dishes were used for each dilution).

### 2.4. Real Sample Preparation

Commercially available infant milk (Humana 3 Pro Balance, produced by Humana Italia S.p.A., Milan, Italy) and whole milk (produced by Assegnatari Associati Arborea s.c.a.p.a., Arborea, Italy) were used. Both products are biologically sourced and sterilized via the ultra-high-temperature (UHT) process. Each milk solution was spiked with *E. coli* at 10^4^ CFU/mL and successively diluted with PBS at two dilution ratios, i.e., 1:10 and 1:20. Sample recoveries were then calculated to evaluate the complex matrix’s influence on the biosensor response.

### 2.5. ELISA Assay Protocol

The antibody titer was evaluated by indirect ELISA test, following the protocol reported by Capo et al. [37]. The *E. coli* RB791 samples, diluted in bicarbonate buffer (50 mM, pH 9.6), were coated on a 96-well plate and incubated at 4 °C overnight. Coating buffer (blank sample), *P. aeruginosa,* and *S. enterica* (10^6^ CFU/mL) were used as controls. After this incubation step, the wells were washed three times with TBS pH 7.4 containing 0.05% Tween-20 (TBS-T) and incubated with 200 μL/well of blocking buffer (5% non-fat dried milk in TBS pH 7.4) at 37 °C for 2 h.

After this step, the wells were rinsed three times, and 100 μL of purified polyclonal antibodies (1 μg/mL diluted in TBS-T) was incubated for 1 h at 37 °C. The plate was rinsed (three times); after that, 100 μL (0.5 μg/mL diluted in TBS-T) of goat anti-mouse IgG-HRP antibody was added, and it was incubated at 37 °C for 1 h. At the end, 50 μL of the enzyme–substrate solution (TMB) was added to each well and incubated at 37 °C. After 10 min, the color development was quenched by adding 50 μL of 2.5 M HCl to each well. The absorbance at 450 nm was then recorded using a Tecan Infinite 200 PRO microplate reader (Tecan Group Ltd., Männedorf, Switzerland).

### 2.6. SPR Sensor Platform Fabrication

The spoon-shaped waveguide, realized in extruded polystyrene (Refractive Index—RI = 1.59 @ 600 nm) and supplied by Italia Soft s.r.l. (Trani, Italy), shows a planar region on the neck and a peculiar concave bowl with eight faces [30].

**Figure 1:**
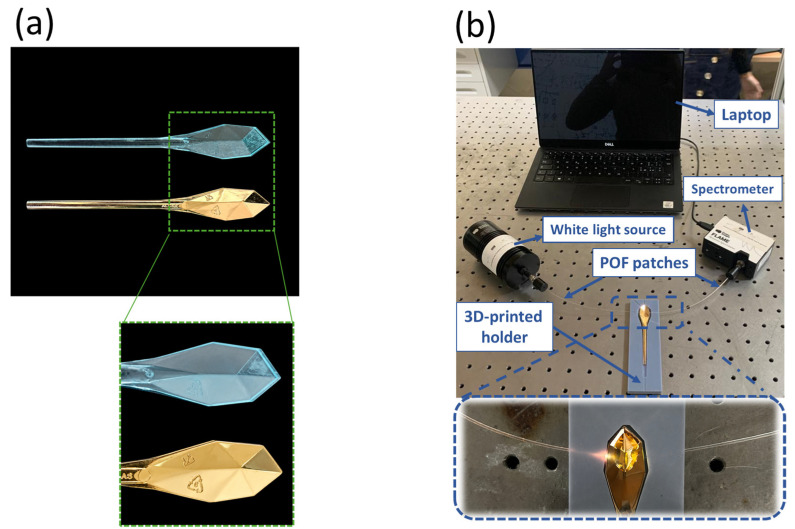
SPR spoon-shaped biosensor. (**a**) Picture of the SPR platform based on a spoon-shaped waveguide, before and after the gold deposition. Inset: Zoom in on the bowl’s sensitive area. (**b**) Image of the experimental setup employed to test the SPR spoon-shaped sensor in orthogonal interrogation mode.

The SPR probe fabrication, based on spoon-shaped waveguides, is extensively described by Cennamo et al. [30]. As shown in Figure 1a, starting with the polymer-based spoon-shaped waveguides, only a gold deposition step is implemented to produce the SPR sensor. The gold film is deposited by a sputter coater machine (Safematic CCU-010, Zizers, Switzerland) via three consecutive steps (20 nm per step) to reach a final thickness equal to about 60 nm. The multi-step process was performed to prevent excessive temperature increases inside the sputtering chamber. Each sputtering step was performed under the following operating conditions: a deposition time of 23 s, current of 60 mA, and working pressure of 0.01 mbar. Moreover, the sputtering process is also monitored via a QCM located in the chamber. It should be emphasized that the sputtering deposition procedure and the compact sputtering chamber dimensions allow a homogenous coating onto the bowl of the spoon-shaped waveguide, ensuring the homogeneity of the gold nanofilm, as shown by the scanning electron microscope (SEM) image reported in Figure S1 (Supplementary Materials file).

Figure 1b shows how the spoon-shaped waveguide is assembled to carry out the optimum plasmonic phenomena in the bowl area, exploiting an orthogonal interrogation mode [30]. The setup consists of two simple components: a halogen lamp as a broad-spectrum light source (HL-2000LL, Ocean Insight, Orlando, FL, USA) and a spectrometer (FLAME-S-VIS-NIR-ES, Ocean Insight, Orlando, FL, USA). Two POF patches (∅ = 1.0 mm) are used to connect the SPR spoon-shaped biochip with the equipment (source and spectrometer).

Moreover, Figure 1b shows the developed 3D-printed holder, produced by a low-cost 3D printer (Photon Mono X UV Resin SLA 3D Printer, Anycubic^®^, Shenzhen, China), which is useful to keep all the components in position (POFs and spoon-shaped waveguide). This custom 3D-printed holder is useful for simply monitoring disposable spoon-shaped biochips. The plasmonic bowl area can be used without microfluid systems, and its peculiar shape represents a built-in measuring cell. This key aspect is suitable for both the functionalization steps to achieve the MRE and the detection of substances of interest via binding tests.

### 2.7. Derivatization and Functionalization of SPR Spoon-Shaped Chip Surface

The bowl area of the SPR spoon-shaped platform was functionalized according to the protocol reported in Arcadio et al. [38]. The functionalization area was chosen for its peculiar characteristics in terms of high optical sensitivity, and the possibility of it acting as a built-in measuring cell [30].

Once the gold nanofilm surface was extensively rinsed with Milli-Q water, it was treated overnight at room temperature with α-lipoic acid (0.3 mM in 8% ethanolic solution). Afterwards, the terminal carboxylic groups of the produced self-assembled monolayer (SAM) were treated for 20 min with EDC/NHS (10 mM/10 mM in MES) at room temperature. Then, the anti-*E. coli* pAb (2.0 mg/mL, 100 µL) was incubated on the activated surface for 2 h in a sealed chamber. Finally, the passivation procedure of the surface was performed by incubating ethanolamine (1 mM in water) for 30 min at room temperature. At the end of the process, the sensor was extensively washed with PBS and stored at 4 °C. The functionalization process was evaluated step by step by recording the SPR wavelength shifts relative to the bare gold surface (gold without the receptor layer), using the same bulk solution (PBS).

### 2.8. SPR Spoon-Shaped Biosensor Measurement Protocol

The dose–response curves were recorded at different concentrations of *E. coli* RB791 (200 μL) and prepared as reported in Section 2.3.

Measurements on the SPR spoon-shaped biosensor were performed by placing 200 μL of the sample on the sensing surface of the bowl and incubating for 10 min at room temperature. The incubation times were defined according to the preliminary binding kinetics experiment reported in Figure S2 (Supplementary Materials file). To remove all bacteria unbound to the antibody, three washing steps of 5 min each were conducted with 200 μL of PBS at pH 7.4 at room temperature. The SPR spectra were collected in the presence of PBS (200 μL) as the bulk solution during the measurements, while the reference spectrum was recorded with air as the surrounding medium, under conditions where SPR did not occur. All SPR spectra were obtained by normalizing the transmitted spectrum in PBS against the reference spectrum.

### 2.9. Statistical Analysis

Raw data were processed and analyzed by using different software. For the bacteria count and indirect ELISA test, statistical analyses were performed with Excel Microsoft 365 (Microsoft Corporation, Redmond, WA, USA). SPR raw data were acquired via proprietary software (Ocean SpectraSuite, version 6.2, Ocean Optics, Orlando, FL, USA), and processed by Matlab software (version R2022b, Mathworks, Natick, MA, USA). The Langmuir fitting of the experimental data was performed by Origin Pro 8.0 software (OriginLab Corporation, Northampton, MA, USA) and the fitting parameters were used to calculate the LoD. Each binding measurement was performed in triplicate in similar working conditions.

## 3. Results and Discussion

### 3.1. Antibody Binding Capability Characterization

The SPR spoon-shaped probe configuration reported in Figure 1 was used to develop a sensitive biosensor for *E. coli* detection. For this purpose, a polyclonal antibody against *E. coli* was purified and characterized before functionalizing the chip surface.

**Figure 2:**
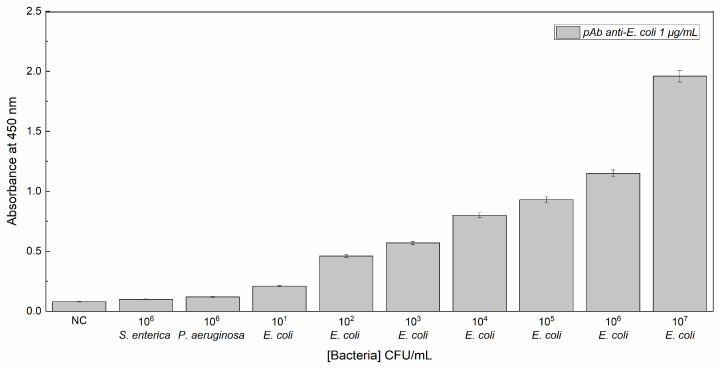
Indirect ELISA results for anti-*E. coli* pAb (1 μg/mL). Binding capability of the produced anti-*E. coli* pAbs (NC: no coating; *S. enterica* and *P. aeruginosa* used as negative controls).

The binding capability and the titer of the purified anti-*E. coli* antibody were evaluated by indirect ELISA experiments. Figure 2 reports the obtained results. The data indicate that the antibodies can bind the *E. coli* adsorbed on the plate surface. A positive response was detected on coated *E. coli* up to 10^1^ CFU/mL. At the same time, no signal was recorded for either non-coated wells or *S. enterica* and *P. aeruginosa* wells. These results demonstrate the specificity of the produced antibody against the *E. coli*.

### 3.2. Derivatization and Functionalization Surface Evaluation

**Figure 3:**
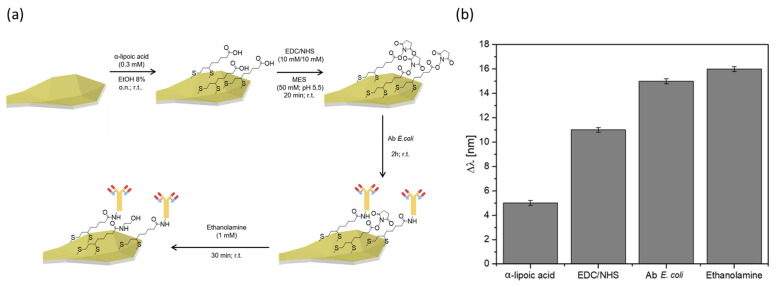
SPR spoon-shaped biosensor fabrication. (**a**) Schematization of the derivatization and functionalization procedure of the SPR spoon-shaped probe with anti-*E. coli* antibodies. (**b**) Variation in the resonance wavelength after each step of the functionalization process (n = 3, standard deviation (SD) = 0.2 nm).

The bowl region of the SPR spoon-shaped probe was functionalized with anti-*E. coli* pAb. More specifically, the SPR spoon-shaped bowl surface was functionalized with pAb according to Arcadio et al. [38]. Figure 3a shows the multi-step functionalization procedure (α-lipoic acid for SAM preparation; activation with EDC/NHS; pAb covalent binding to the SAM and passivation procedure). The achievement of each functionalization step was monitored by the changes in the SPR wavelength (∆λ), calculated with respect to the value obtained for a bare platform (surface without receptor), and considering PBS as the surrounding medium.

Figure 3b shows the variation in the resonance wavelength (Δλ_max_), calculated with respect to the value of the bare chip, after each functionalization step. In particular, the data show an increase in the refractive index at the gold–dielectric interface with respect to the bare surface (using PBS as bulk) due to the derivatization and functionalization procedure. The obtained red-shift variation in the resonance wavelength confirms the effectiveness of the immobilization process.

### 3.3. Ab-SPR Spoon-Shaped Sensor Response in E. coli Detection

The SPR biosensor response to *E. coli* was tested in different matrices, PBS and skim milk for *E. coli* RB791, in a concentration ranging from 10^6^ to 10^1^ CFU/mL. Moreover, *E. coli* ATCC 11228 has been tested in LB medium in the same concentration range. These tests have been carried out to study the interaction between the *E. coli* membrane protein and the bioreceptor layer, varying both the matrix and the *E. coli* strain.

**Figure 4:**
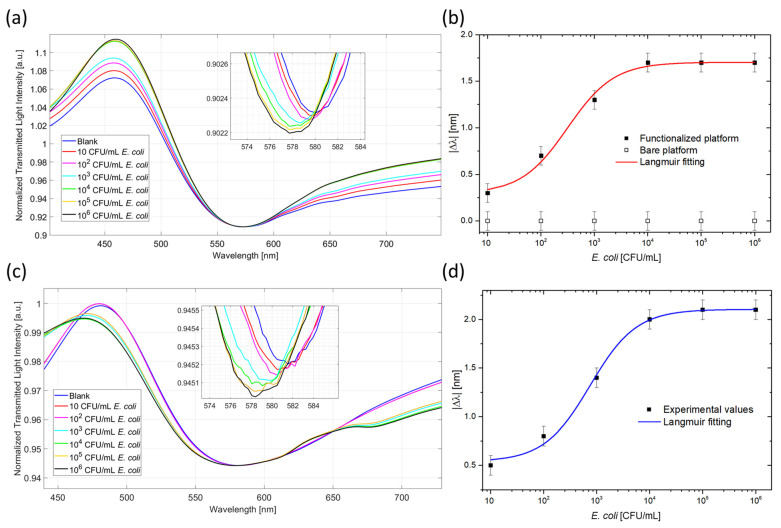
Performance evaluation of the SPR spoon-shaped biosensor to *E. coli* RB791 in PBS (**a**,**b**) and skim milk (**c**,**d**). (**a**) SPR spectra relative to *E. coli* RB791 detection at increasing numbers of bacteria diluted in PBS. (**b**) Absolute SPR wavelength shift value (phenomena around 580 nm), calculated with respect to the blank, as a function of *E. coli* RB791 concentration in PBS for the bare platform (□) and the functionalized platform (■). The experimental data are shown on a semi-logarithmic scale, along with the Langmuir fit (red line) for the functionalized platform. (**c**) SPR spectra relative to *E. coli* RB791 detection with an increasing number of bacteria diluted in skim milk (diluted at 5% in PBS). (**d**) Absolute SPR wavelength shift value (phenomena around 580 nm), calculated with respect to the blank, as a function of *E. coli* RB791 bacteria in diluted skim milk for the functionalized platform (■). The experimental data are shown on a semi-logarithmic scale, along with the Langmuir fitting (blue line). The shown error bar (**b**,**d**) represents the standard deviation of three measurements (n = 3).

Figure 4a reports the SPR spectra obtained in PBS for different *E. coli* RB791 concentrations. The SPR spectra are obtained after 10 min of sample incubation and a washing step (as described in Section 2.8). The spectra were obtained by normalizing the acquired transmitted spectra in PBS to the reference spectrum (the air spectrum). A decrease in resonance wavelength (resonance wavelength around 580 nm) was observed at increased *E. coli* RB791 concentrations. This wavelength shift is associated with the binding of the anti-*E. coli* pAb with the analyte, suggesting that the RI of the recognizing layer in contact with the gold surface decreases after the binding. In other words, the anti-*E. coli* pAb binding with the analyte induces a conformational change in the bioreceptor layer associated with a decrease in the measured refractive index at the SPR sensing interface. This phenomenon ultimately results in a shift in the SPR peak toward lower wavelengths (blue shift). This behavior can be attributed to a more efficient conformational reorganization of the receptor layer following binding, and it has already been observed in previous studies in which the same spoon-shaped waveguide was coupled to different protein-based receptors [38].

As a control, a bare platform was tested with the same bacteria concentration range, and the data show no resonance shift in the presence of increasing concentrations of *E. coli* RB791 (Figure 4b). Moreover, Figure 4b reports the biosensor’s dose–response curve obtained by fitting the experimental data with the Langmuir model equation, which has the general formula reported in Equation (1).

(1) $$\left|\Delta \lambda \right|=\left|\lambda_{c}-\lambda_{0}\right|=\left|\Delta \lambda_{max}\right|\cdot \left(\frac{c}{K+c}\right)$$

where λ_c_ is the resonance wavelength at the analyte concentration c; λ_0_ is the resonance wavelength in the absence of the analyte (blank); Δλ_max_ is the maximum value of Δλ, calculated by subtracting the blank value from the saturation value; and K is the Langmuir fitting constant. The obtained fitting parameters are listed in Supplementary Table S1.

The biosensor response to *E. coli* RB791 was also tested in skim milk (diluted at 5% in PBS), using the same bacteria concentration range, to evaluate the matrix effect on the biosensor response. As for the PBS measurements, increasing *E. coli* concentrations in skim milk produces a decrease in resonance wavelength (Figure 4c). Figure 4c and 4d show the SPR spectra registered in skim milk and the related binding isotherm with the Langmuir fitting, respectively. It should be noted that the SPR spectra reported in Figure 4a,c were shifted by a pure translation in the *y*-axis direction, in order to compare the SPR spectra’s minimums (resonance wavelength around 580 nm), to determine the resonance wavelength, which is considered the biosensor output.

The fitting parameters obtained by the Langmuir model can be used to calculate the analytical parameters of the biosensor for *E. coli* detection in terms of sensitivity at low concentration (S_lowc_), LoD, and affinity constant (K_aff_). In particular, the S_lowc_ can be calculated as reported in Equation (2):

(2) $$S_{lowc}=|\Delta \lambda_{max}|/K$$

Instead, the LoD can be estimated as the ratio between three times the standard deviation of the blank (SD_λ0_) and the sensitivity at low concentration (S_lowc_) [39], as reported in Equation (3):

(3) $$LoD=(3\times {SD}_{\lambda_{0}})/S_{lowc}$$

Finally, the affinity constant can be obtained as reported in Equation (4):

(4) $$K_{aff}=1/K$$

The calculated variables reported in Supplementary Table S1 were used to evaluate the sensor parameters mentioned above with respect to *E. coli* RB791 detection in PBS and skim milk, reported in Table 1.

The calculated LoDs for the PBS and 5% skim milk solution were 6.5 and 6.8 CFU/mL, respectively. The obtained parameters were comparable and supported the possibility of employing the Ab-SPR spoon-shaped biosensor for monitoring *E. coli* in skim milk, with negligible matrix effects under this dilution condition.

Similarly, additional tests were carried out with a different strain of *E. coli*, namely ATCC 11228, prepared in LB medium and diluted in PBS within the same concentration range as above. As shown in Figure S3, the results, as described in the performance parameters reported in Table S3 (calculated from the fitting parameters reported in Table S2), were highly comparable across different strains of *E. coli*. This result demonstrates that the proposed SPR biosensor response is independent of the *E. coli* strain, due to the high selectivity between its membrane protein and the bioreceptor layer.

It should also be emphasized that, as observed in the experimental measurements, the increased light scattering in the bowl-sensitive area allowed monitoring of the interaction between a bioreceptor and large-sized analytes, such as *E. coli* bacteria, at visible wavelengths.

Furthermore, the obtained LoD values compared with other sensors presented in the literature are reported in Table 2. As shown in Table 2, the proposed sensing approach demonstrated an improvement in the performance of *E. coli* detection by exploiting SPR spoon-shaped biosensor configurations. Beyond the analytical performance, the proposed SPR spoon-shaped biosensor presents several practical and technological advantages. The concave bowl region acts as an integrated measuring cell, without the need for external microfluidic systems. This feature simplifies experimental configuration, reduces the number of required components, and makes the platform particularly suitable for rapid, on-site analyses. In addition, the sensor is based on a simple polymeric waveguide fabricated via gold sputtering, enabling low-cost fabrication and disposable use.

### 3.4. Selectivity Tests

**Figure 5:**
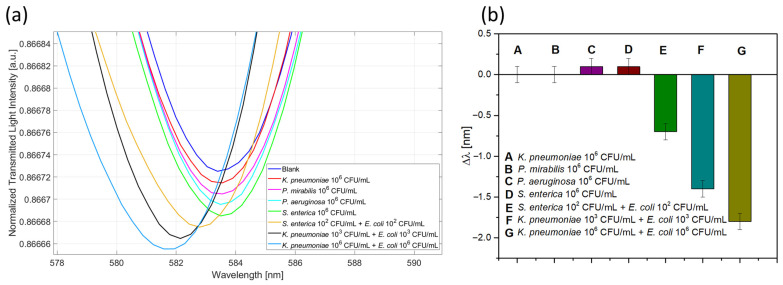
(**a**) SPR spectra achieved via the SPR spoon-shaped biosensor towards interfering bacteria (*K. pneumoniae*, *P. mirabilis*, *P. aeruginosa* and *S. enterica*) taken singularly and in mixed cultures with *E. coli*. (**b**) Resonance wavelength shift (Δλ) calculated with respect to the blank for *K. pneumoniae*, *P. mirabilis*, *P. aeruginosa* and *S. enterica* and mixed cultures with *E. coli*. The error bars were calculated as the standard deviation of the dataset (n = 3).

Two different experimental controls were performed to test the selectivity of the developed biosensor. In the first test, the response of the SPR probe combined with anti-*E. coli* pAb was monitored in the presence of an interfering bacterium, i.e., *P. aeruginosa*, and other bacteria belonging to *Enterobacteriaceae* species, namely *S. enterica*, *K. pneumoniae* and *P. mirabilis,* each of which was considered singularly at 10^6^ CFU/mL. Furthermore, to better mimic the complexity of real samples, other selectivity assays were performed using mixed cultures: *E. coli*/*S. enterica,* each of which were at 10^2^ CFU/mL, and two different solutions of *E. coli*/*K. pneumoniae,* each at 10^3^ CFU/mL and 10^6^ CFU/mL, respectively. Figure 5a reports the SPR spectra obtained with the four interfering bacteria considered individually and in mixed cultures. In contrast, Figure 5b summarizes the SPR biosensor response in terms of Δλ achieved during the different selectivity tests. As shown in Figure 5, the interfering bacteria considered individually did not produce any resonance wavelength shift, in contrast to what was observed with mixed cultures, where the presence of *E. coli* led to a clear blue shift, confirming the specificity of the system. It should also be stressed that the Δλ values in mixed-culture tests were highly comparable to those obtained from dose–response curves with a solution containing *E. coli* only (see Figure 4).

It should also be stressed that *P. aeruginosa* was not included in mixed-culture experiments, as this species can establish strong antagonistic or cooperative interactions with coexisting microorganisms, potentially affecting their growth and altering the microbial composition of the culture [41,42]. Such interspecies interactions could bias the interpretation of selectivity results by introducing growth-inhibition effects unrelated to the assay’s analytical performance.

In the second test, the specificity of the system was evaluated by testing an SPR spoon-shaped platform functionalized with a different antibody, with *E. coli*. For this purpose, the surface was functionalized with a different antibody, the pAb raised against the classical swine fever virus (anti-CSFV), and the biosensor was tested with increasing *E. coli* concentrations. The obtained data are reported in Figure S4 (Supplementary Materials file). The SPR spectra obtained do not show resonance wavelength shifts, excluding non-specific binding of *E. coli* on the surface.

### 3.5. Tests in Real Samples

**Figure 6:**
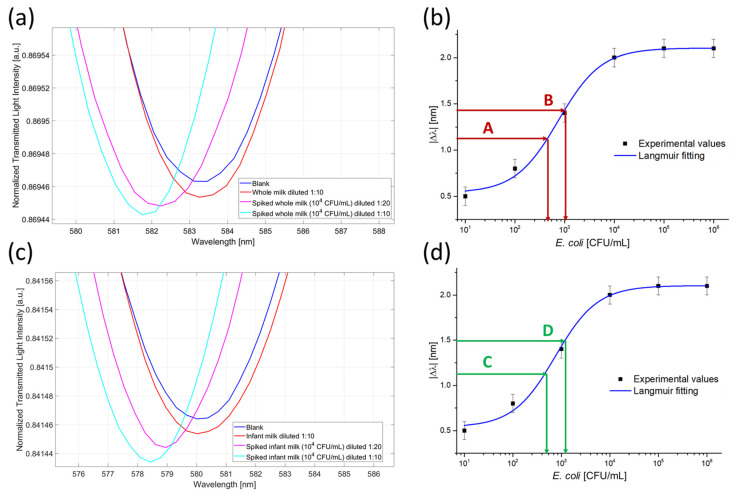
Biosensor response with *E. coli* spiked in real samples: SPR spectra relative to (**a**) whole milk and (**c**) infant milk diluted samples; graphical estimation of the *E. coli* concentration from the dose–response curve for (**b**) whole milk and (**d**) infant milk samples at the corresponding dilution ratios.

To determine the SPR spoon-shaped biosensor response to more complex matrices, experimental tests on commercial whole milk and infant milk were carried out. Both milk samples were prepared as described in Section 2.4. To summarize, whole milk and infant milk were both spiked with *E. coli* at 10^4^ CFU/mL and then diluted 1:20 and 1:10 with PBS. In a preliminary phase, for each sample, non-spiked milk solutions diluted 1:10 were tested to evaluate possible non-specific deposition on the gold surface due to the matrix complexity. As shown in Figure 6a and 6c, negligible Δλ was achieved for both non-spiked whole milk and infant milk samples, respectively. In Figure 6a,c, the results for the spiked samples are also reported. In order to graphically estimate the *E. coli* concentration in each milk sample, as reported in Figure 6b,d, the Δλ values produced by the diluted milk samples can be used together with the dose–response curve (calibration curve reported in Figure 4d).

Table 3 summarizes the achieved shifts for each diluted sample, the dilution ratio, and the graphical estimate of *E. coli* concentration (obtained as shown in Figure 6). In particular, the *E. coli* concentration spiked in both milk samples can be estimated by multiplying the concentration reported in Table 3, achieved from the dose–response curve and the diluted sample response, by the corresponding dilution factor.

For each sample, two dilution ratios were considered for redundancy in the final concentration estimation, which was calculated as the mean of these two values. In such a way, the recovery of the measured samples was 95% for the whole milk sample and 102% for the infant milk sample. Considering these recovery values, the matrix effect under these working conditions can be considered insignificant.

## 4. Conclusions

In this work, an SPR spoon-shaped biosensor to detect *E. coli* was developed and tested. The obtained results showed specificity against interfering bacteria (both singularly and in a mixed culture with *E. coli*) and the potential to achieve a remarkable LoD (6.8 CFU/mL).

The developed biosensor was tested on commercial milk samples to assess its suitability for real-world scenarios. The achieved recovery values (95% for whole milk and 102% for infant milk) confirmed a negligible matrix effect under the considered working conditions.

The proposed biosensor offers additional advantages, including minimal sample volume and a rapid response time (10 min), without the need for microfluid systems (the spoon-shaped geometry of the optical waveguide integrates the sensing region and the measuring cell into a single disposable optical element). Furthermore, the possibility of analyzing the sample without any pre-treatment or culture pre-enrichment makes it suitable for real-time, selective monitoring of *E. coli* in the field or as a POCT.

## Figures and Tables

**Table 1:** Biosensor parameters relative to *E. coli* RB791 detection in PBS and 5% skim milk.

	S_lowc_ [nm/(CFU mL^−1^)]	LoD [CFU/mL]	K_aff_ [mL/CFU]
PBS	4.6 × 10^−3^	6.5	2.7 × 10^−3^
5% Skim Milk	4.4 × 10^−3^	6.8	2.3 × 10^−3^

**Table 2:** Comparative analysis of SPR biosensors for *E. coli* detection in terms of LoDs in different matrices.

Sensing Technologies	Matrix	LoD [CFU/mL]	Reference
SPR bioanalyzer combined with microfluidic cell	-	1.87 × 10^3^	[22]
LSPR platform based on gold nanorod array	Water	8.4	[27]
SPR optical fiber sensor modified with biofunctionalized MoS_2_ nanosheets	PBS	94	[28]
SPR optical fiber sensor based on silver nanoparticle-reduced graphene oxide covered by a gold film	Water	5 × 10^2^	[29]
SPR imaging based on a nanohole array-based sensor and CMOS detector	Urine	100	[40]
SPR spoon-shaped biosensor	PBS	6.5	This work
SPR spoon-shaped biosensor	Skim milk 5%	6.8	This work

**Table 3:** Summary of the biosensor response to spiked whole milk and infant milk samples, tested at different dilution factors, and graphical estimation of the *E. coli* concentration via the dose–response curve (Figure 6).

Sample	Label	Dilution Factor	|Δλ| [nm]	Estimated *E. coli* Concentration of the Diluted Sample [CFU/mL]	Estimated *E. coli* Concentration Sample [CFU/mL]	Recovery
Whole milk spiked with *E. coli* 10^4^ CFU/mL	A	1:20	1.1	4.7 × 10^2^	(4.7 × 10^2^) × 20 = 9.4 × 10^3^	95%
	B	1:10	1.4	9.6 × 10^2^	(9.6 × 10^2^) × 10 = 9.6 × 10^3^	
Infant milk spiked with *E. coli* 10^4^ CFU/mL	C	1:20	1.1	4.7 × 10^2^	(4.7 × 10^2^) × 20 = 9.4 × 10^3^	102%
	D	1:10	1.5	1.1 × 10^3^	(1.1 × 10^3^) × 10 = 1.1 × 10^4^	

## Data Availability

The original contributions presented in this study are included in the article/Supplementary Material. Further inquiries can be directed to the corresponding author.

## Supplementary Materials

The following supporting information can be downloaded at https://www.mdpi.com/article/10.3390/bios16070371/s1: Figure S1. Scanning electron microscope image of the bowl of the SPR spoon-shaped biosensor. Inset: Zoom into a sensitive area of the bowl. Figure S2. Ab-SPR spoon-shaped biosensor response over time. Ab-SPR spoon-shaped sensor SPR spectra, monitored at different incubation times with 10^2^ CFU/mL of *E. coli* RB791. The measurements were performed at 25 °C. Table S1. Langmuir fitting parameters relative to *E. coli* RB791 detection in PBS and 5% skim milk. Figure S3. (a) SPR spectra achieved via Ab-SPR spoon-shaped biosensor at *E. coli* ATCC 11228 concentrations ranging from 10^6^ to 10^1^ CFU/mL. (b) Dose–response curve achieved in *E. coli* ATCC 11228 detection. Table S2. Langmuir fitting parameters relative to *E. coli* ATCC 11228 detection. Table S3. Biosensor parameters relative to *E. coli* ATCC 11228 detection. Figure S4. SPR spectra at different concentrations of *E. coli* (ranging from 10^6^ to 10^1^ CFU/mL) tested on an SPR spoon-shaped platform functionalized with a different pAb not specific for *E. coli*. The measurements were performed at 25 °C.
