# Supplementary material for: Biosensors Based on Plasmonic Spoon-Shaped Platforms as a Point-of-Care Tool for Escherichia coli Detection
Source: Biosensors (Basel). 2026 Jul 8;16(7):371. doi: 10.3390/bios16070371 (PMC13407397; doi:10.3390/bios16070371)
Supplement: Supplementary file 1 [file biosensors-16-00371-s001.zip › biosensors-4344673-supplementary.pdf]

Supplementary Materials

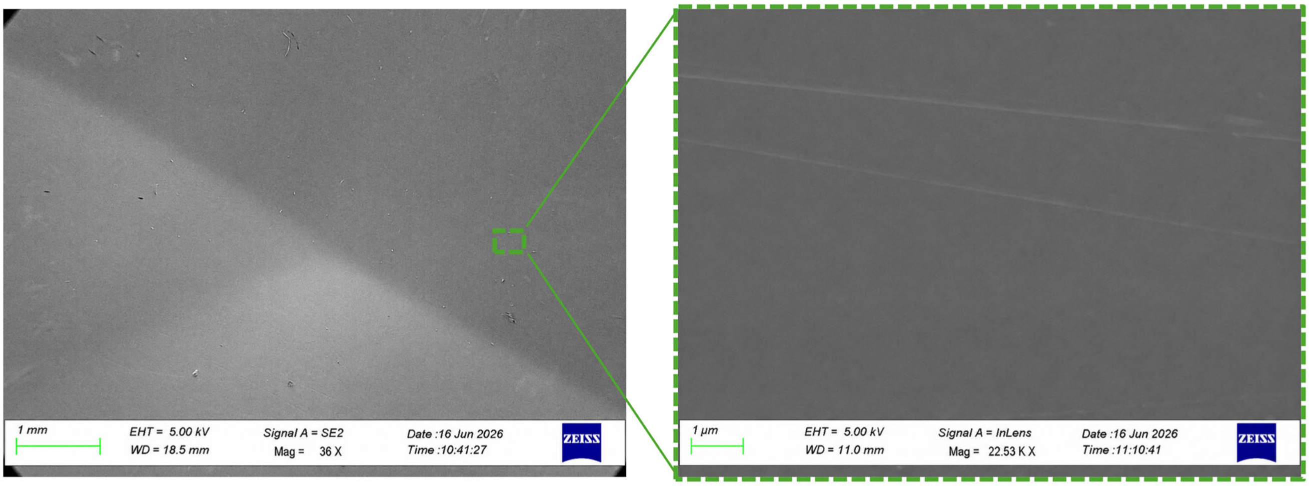

Figure S1. Scanning electron microscope image of the bowl of the SPR spoon-shaped biosensor. Inset: zoom into a sensitive area of the bowl.

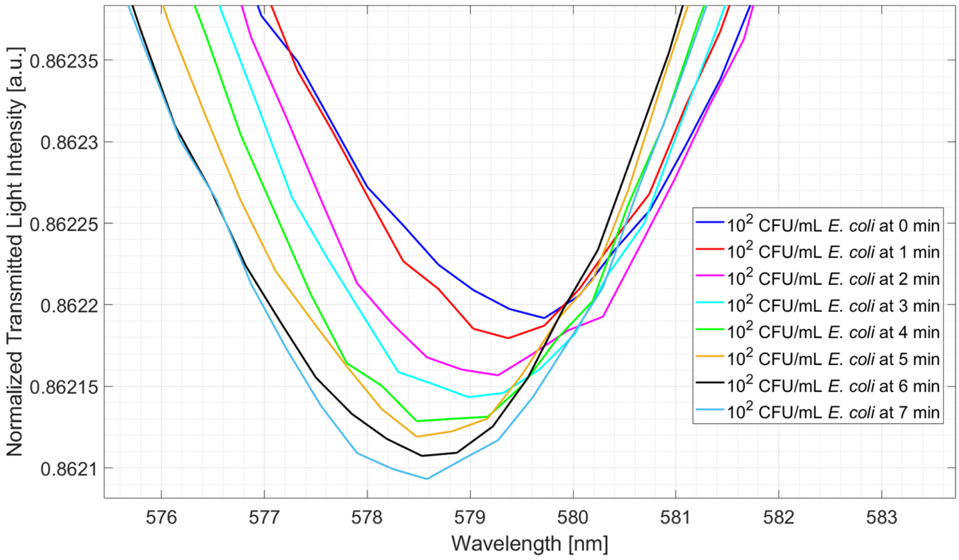

Figure S2. Ab-SPR spoon-shaped biosensor response over time. Ab-SPR spoon-shaped sensor SPR spectra, monitored at different incubation times with 10^2 CFU/mL of *E. coli* RB791. The measurements were performed at 25 °C.

Table S1. Langmuir fitting parameters relative to *E. coli* RB791 detection in PBS and skim milk 5%.

Matrix	$\lvert\lambda_0\rvert$ [nm]	$\lvert\Delta\lambda_{max}\rvert$ [nm]	K [CFU/mL]	Statistics	
				χ^2	R^2
**PBS**	0.27 ± 0.01	1.71 ± 0.02	374 ± 43	0.093	0.998
**Skim milk 5%**	0.41 ± 0.01	1.91 ± 0.02	431 ± 77	0.077	0.998

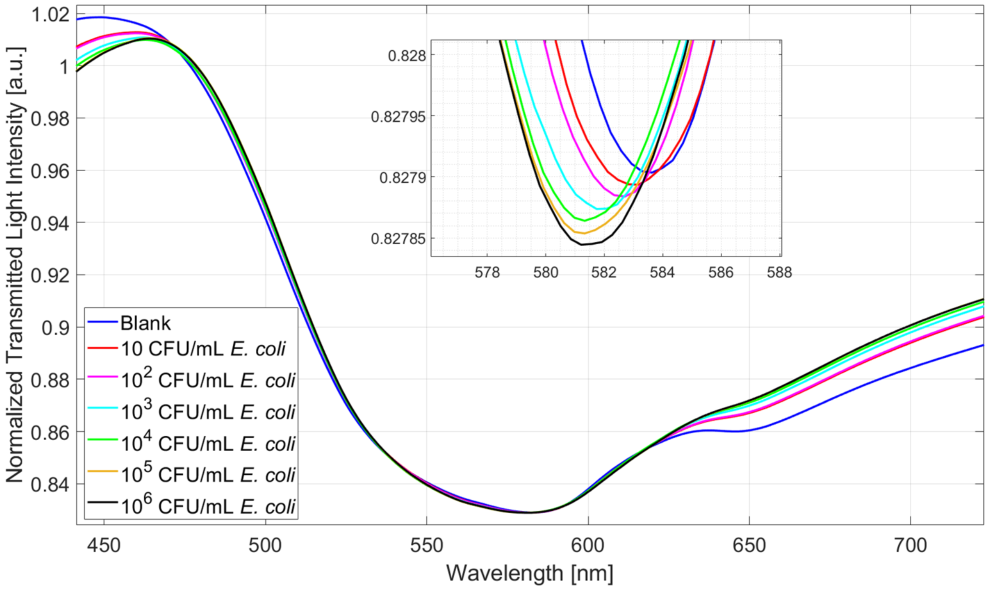

(**a**)

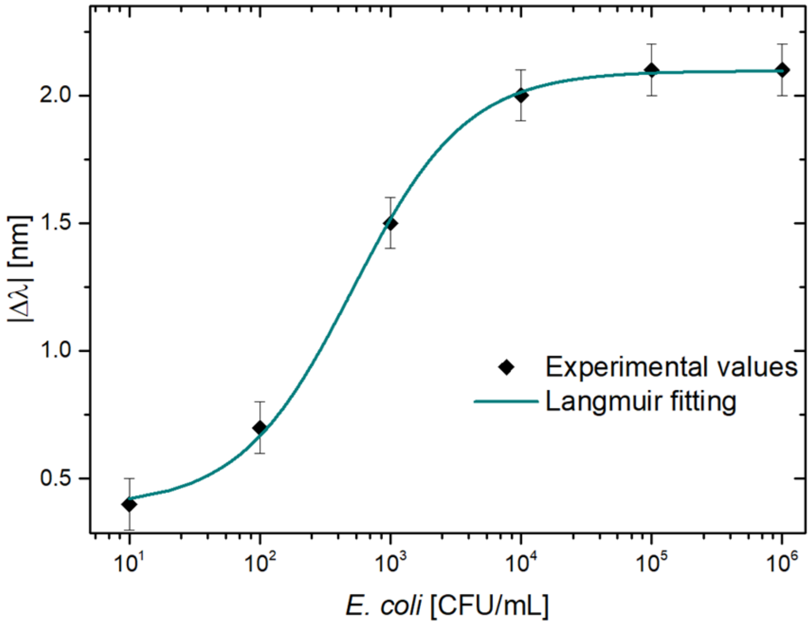

(**b**)

Figure S3. (**a**) SPR spectra achieved via the SPR spoon-shaped biosensor at *E. coli* ATCC 11228 concentrations ranging from 10^6 to 10^1 CFU/mL. (**b**) Dose-response curve achieved in *E. coli* ATCC 11228 detection.

Table S2. Langmuir fitting parameters relative to *E. coli* ATCC 11228 detection.

**$\lvert\lambda_0\rvert$ [nm]**	**$\lvert\Delta\lambda_{max}\rvert$ [nm]**	**K [CFU/mL]**	**Statistics**	
			**χ^2**	**R^2**
0.38 ± 0.02	2.09 ± 0.01	515 ± 42	0.072	0.999

Table S3. Biosensor parameters relative to *E. coli* ATCC 11228 detection.

**S_{lowc} [nm/(CFU mL^{-1})]**	**LoD [CFU/mL]**	**K_{aff} [mL/CFU]**
4.1×10^{-3}	7.2	2.1×10^{-3}

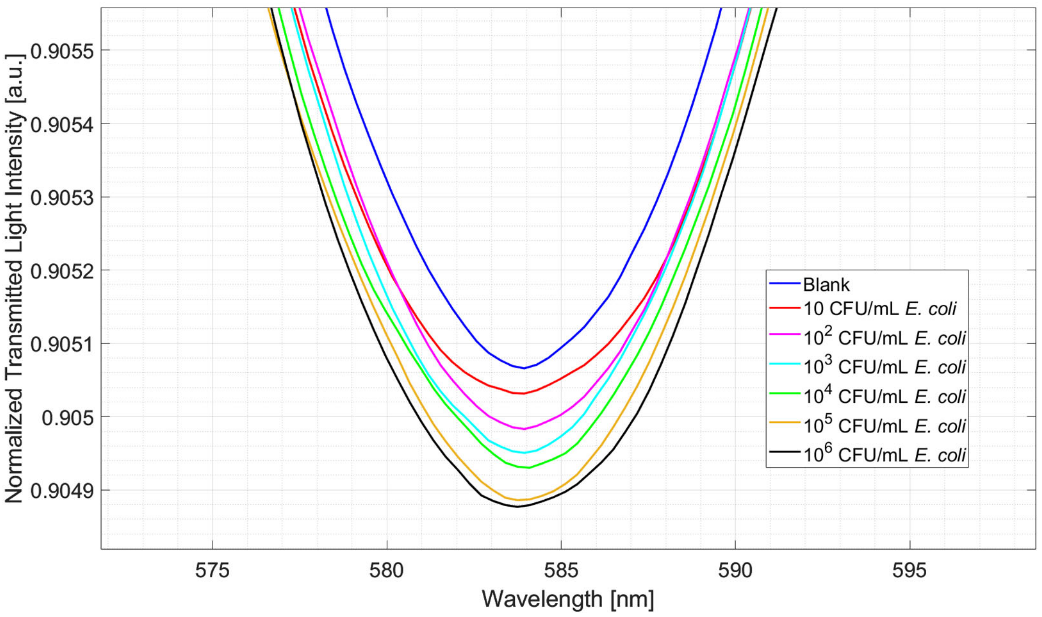

Figure S4. SPR spectra at different concentrations of *E. coli* (ranging from 10^6 to 10^1 CFU/mL) tested on an SPR-spoon-shaped platform functionalized with a different pAb not specific for *E. coli*. The measurements were performed at 25 °C.
